# Validation of an instrumented dummy to assess mechanical aspects of discomfort during load carriage

**DOI:** 10.1371/journal.pone.0180069

**Published:** 2017-06-29

**Authors:** Patrick D. Wettenschwiler, Simon Annaheim, Silvio Lorenzetti, Stephen J. Ferguson, Rolf Stämpfli, Agnes Psikuta, René M. Rossi

**Affiliations:** 1 Empa, Swiss Federal Laboratories for Materials Science and Technology, St. Gallen, Switzerland; 2 Institute for Biomechanics, ETH Zurich, Zurich, Switzerland; Virginia Tech, UNITED STATES

## Abstract

Due to the increasing load in backpacks and other load carriage systems over the last decades, load carriage system designs have to be adapted accordingly to minimize discomfort and to reduce the risk of injury. As subject studies are labor-intensive and include further challenges such as intra-subject and inter-subject variability, we aimed to validate an instrumented dummy as an objective laboratory tool to assess the mechanical aspects of discomfort. The validation of the instrumented dummy was conducted by comparison with a recent subject study. The mechanical parameters that characterize the static and dynamic interaction between backpack and body during different backpack settings were compared. The second aim was to investigate whether high predictive power (coefficient of determination R^2^>0.5) in assessing the discomfort of load carriage systems could be reached using the instrumented dummy. Measurements were conducted under static conditions, simulating upright standing, and dynamic conditions, simulating level walking. Twelve different configurations of a typical load carriage system, a commercially available backpack with a hip belt, were assessed. The mechanical parameters were measured in the shoulder and the hip region of the dummy and consisted of average pressure, peak pressure, strap force and relative motion between the system and the body. The twelve configurations consisted of three different weights (15kg, 20kg, and 25kg), combined with four different hip belt tensions (30N, 60N, 90N, and 120N). Through the significant (p<0.05) correlation of the mechanical parameters measured on the dummy with the corresponding values of the subject study, the dummy was validated for all static measurements and for dynamic measurements in the hip region to accurately simulate the interaction between the human body and the load carriage system. Multiple linear regressions with the mechanical parameters measured on the dummy as independent variables and the corresponding subjective discomfort scores from the subject study as the dependent variable revealed a high predictive power of the instrumented dummy. The dummy can explain 75% or more of the variance in discomfort using average pressures as predictors and even 79% or more of the variance in discomfort using strap forces as predictors. Use of the dummy enables objective, fast, and iterative assessments of load carriage systems and therefore reduces the need for labor-intensive subject studies in order to decrease the mechanical aspects of discomfort during load carriage.

## Introduction

During the last decades, the loads carried in backpacks and other load carriage systems have increased [1, 2]. As a consequence, load carriage system designs have to be adapted accordingly to minimize discomfort and to reduce the risk of injury. Discomfort during load carriage is a major issue relevant for e.g. infantry [3–6], school children’s or student’s load carriage [7–10], and outdoor activities [11–13]. The importance of discomfort is further underlined by its influence on user acceptance [14, 15]. Recently, a growing interest has been shown in the mechanical aspects of discomfort during load carriage: Piscione et al. [16] analyzed the effect of mechanical compression on shoulder muscle fatigue and Hadid et al. [17] modeled the mechanical strains and stresses in the soft tissue of the shoulder. Older publications by Holloway et al. [18] and Sangeorzan et al. [19] already investigated the relationship between external pressure loading and skin blood flow. Furthermore, a number of investigations reported a relationship between discomfort and the external pressure loading occurring during sitting activities, e.g. in car seats [20], or office chairs [21]. While most of these studies investigated static scenarios, the dynamic aspects of the mechanical loading may well play an important role for the perceived discomfort. There are still many unsolved questions regarding the mechanical aspects of discomfort.

Currently, assessing the mechanical aspects of discomfort in load carriage systems is mostly done using subject studies, where the participants provide subjectively perceived discomfort scores. However, these studies are labor-intensive and include further challenges, such as intra-subject and inter-subject variability or ethical considerations for extreme applications. Furthermore, direct comparisons of load carriage systems are often only possible within the same study. Hence, to avoid biased results, an objective measurement tool in a laboratory environment would be preferable, especially when comparing many different load carriage systems. Such a tool would also be beneficial for the industry during different stages of the development of load carriage systems. A first instrumented physical model, developed for this purpose, was presented by Stevenson et al. [5], measuring average pressure, peak pressure, strap forces and relative motion between load carriage system and body in different body regions. For the shoulder region, their model explained 31% of the variation in discomfort using average pressure measured on the model [22]. As a validation, they presented significant correlations (p<0.05) between the mechanical parameters measured on their model and discomfort scores gathered from subjects [22]. For further development in this field, future models need to enhance the predictive power in assessing discomfort, while at the same time providing a solid model validation. Recently, new possibilities for the validation of such models have opened up. For the first time, mechanical parameters that characterize the static and dynamic interaction between a load carriage system and the human body and the subjectively perceived discomfort, have been measured in a subject study [23]: A typical load carriage system, a commercially available backpack with a hip belt, was tested on ten male subjects during level walking using twelve different configurations, varying in load weight and hip belt tension. This study revealed that static peak pressure, or alternatively strap force in the shoulder strap and the hip belt, can account for more than 85% of the variation in subjectively perceived discomfort in the shoulder and the hip region [23]. As a consequence, a physical model that is able to accurately simulate human load carriage, regarding the relevant mechanical parameters, would be a promising tool to assess the mechanical aspects of discomfort of load carriage systems economically, objectively and reliably. Therefore, the first aim of this study was to validate a newly built instrumented dummy by comparing mechanical parameters that characterize the static and dynamic interaction between load carriage system and body, during different backpack settings, with the recent subject study [23]. The second aim of this study was to investigate whether a high predictive power (coefficient of determination R^2^>0.5) in assessing the discomfort of load carriage systems can be reached using the instrumented dummy.

## Materials and methods

### Instrumented dummy

**Fig 1:**
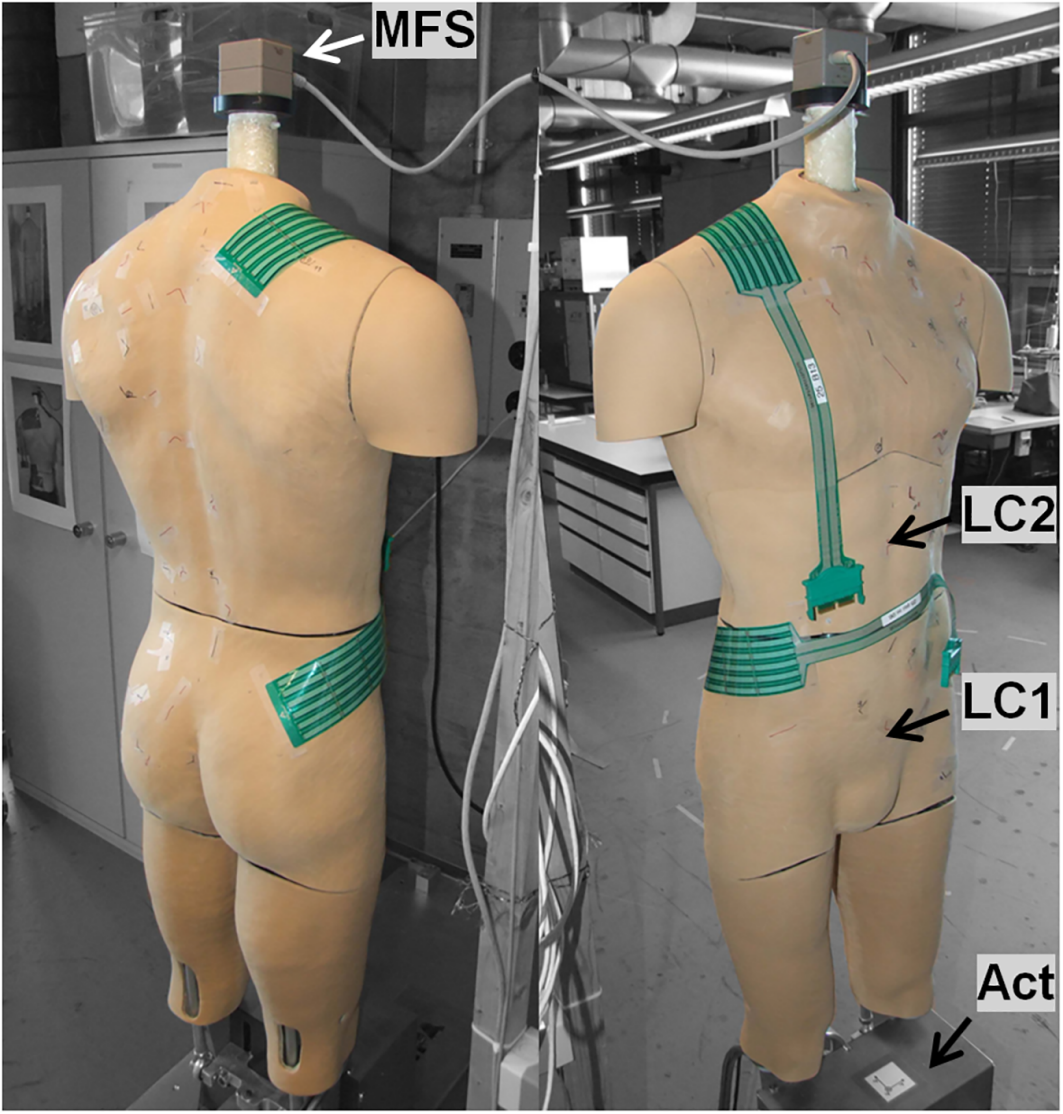
Newly built dummy. Tekscan type 9811E sensors are mounted in the shoulder and the hip region. MFS: magnetic field source of the 3D motion tracking device; LC1: load cell in the pelvis; LC2: load cell in the upper body; Act: actuator for the sinusoidal motion.

Based on the model of Stevenson et al. [5], we constructed a dummy to assess mechanical aspects of discomfort. It features a male anatomy with a chest circumference of 104cm, a waist circumference of 81cm, and a waist-to-neck length of 52cm (Fig 1). Anthropometrics were based on the body dimensions of a Canadian Forces 50^th^ percentile male soldier, calculated using segment properties defined by Winter [24]. As a skin analogue, its body surface consists of a 3.0mm thick layer of nora® Lunasoft SLW (Otto Bock Healthcare, Duderstadt, Germany). This closed cell foam was developed for the interface of human prostheses and has a Shore A hardness of 30, complying with the hardness of human skin and the subcutaneous tissue [25]. To compensate the external loading of the backpack, the natural forward leaning that occurs during posterior load carriage [26–29] is replicated in the dummy. According to Grimmer et al. [26], the flexion during posterior load carriage occurs in the ankle. Therefore, in this study, the forward lean angle in the upper ankle joint is calculated to balance the moment about the upper ankle joint. For this calculation, the mass distribution of the human body segments of a medium sized male (bodyweight 81.5kg, size 178.4cm) was applied according to Armstrong et al. [30]. The mass (± 0.1kg) and the center of mass (± 0.1cm) of the load carriage system in relation to the upper ankle joint are measured using a trifiliar pendulum and a custom made straightedge in the sagittal plane of the dummy. The three different masses of the load carriage system used in this study (see section ‘Load Carriage System’ below) required forward leaning angles of the dummy of 2.10°, 2.64°, and 3.13°, respectively, to achieve equilibrium. For practical reasons, the dummy did not wear clothes. Clothes were shown to have only a minor influence on the pressure distribution on the body surface during load carriage [31]. For the static scenario, the dummy simulates upright standing, while for the dynamic scenario the dummy simulates walking or jogging through a vertical sinusoidal motion. Stride frequency and vertical displacement were obtained during walking [32–35]. The ranges found for walking speed between 0.56m/s and 1.67m/s provided a stride frequency between 0.51Hz and 1.04Hz and a vertical displacement between 0m and 0.045m. The frequency chosen for dynamic testing in this study was 1.77Hz, which corresponds to a stride frequency of 0.98Hz. or 1.25m/s [32]. The amplitude of the vertical sinusoidal motion in this study was 0.0508m as this was the value chosen by Stevenson et al. [5] and the dummy presented in this study was built based on their model. Theoretically, the dummy can also measure the load distribution between the hip and the upper body during load carriage. However, for this study, the two six degrees of freedom load cells, which were integrated into the dummy for this purpose, were not involved in the measurements. They may serve in future studies, e.g. to analyze the loading of the lumbar spine during load carriage.

### Load carriage system

**Fig 2:**
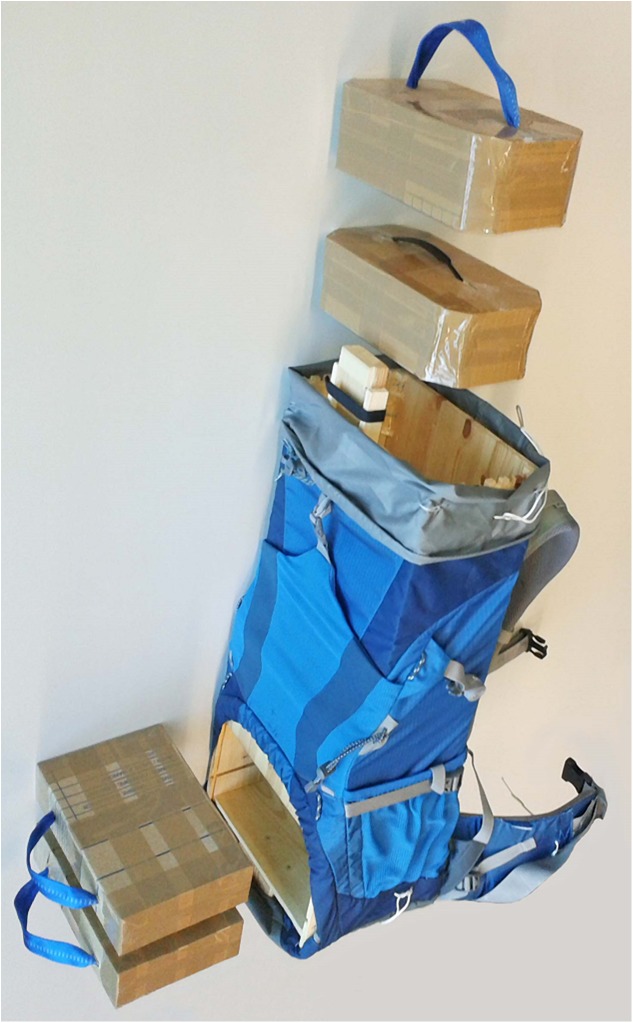
Load carriage system. Cardboard boxes filled with sand could be inserted through an opening in the backpack and the wooden box at the bottom and the top to enable efficient change of the payload, while keeping the center of mass in the same position.

The commercially available backpack “Deuter ACT Lite 50+10” (Deuter Sport GmbH, Gersthofen, Germany) served as load carriage system in this study. Its intended use ranges from travelling to trekking and alpine tours. Due to the use of the electromagnetic tracking system (see section ‘Relative Motion Measurement’ below), no metal parts were allowed in the load carriage system. Even the paramagnetic aluminum rods that formed the frame in the back wall of the backpack had to be removed. Instead, a wooden box (65.0cm height, 27.5cm length, 15.8cm depth, and 0.9cm wall thickness) was inserted into the backpack and rigidly fixed to the back wall, replacing the function of the aluminum rods and thus enabling a load transfer between the hip belt and the shoulder straps. Rather than applying several different load carriage systems, we applied twelve different configurations of this one typical load carriage system, thus eliminating potential effects of the design on discomfort. As load weight and hip belt tension have the largest effects on interface pressure and strap forces [36], the twelve configurations used in our study consisted of a combination of three different loads and four different hip belt tensions: The hip belt lengths were adjusted to reach 30N, 60N, 90N, and 120N (±1N) of static tension on the dummy. The total masses of the load carriage system configurations were 15.0kg, 20.0kg, and 25.0kg. For an efficient change of the payload, cardboard boxes filled with sand could be inserted through an opening in the backpack and the wooden box at the bottom and the top (Fig 2). All configurations respected lateral symmetry, while the center of mass was positioned 30.5cm from the bottom in all configurations.

### Measurements

#### Body surface pressure measurement

The pressure on dedicated regions of the surface of the dummy was recorded using Tekscan type 9811E pressure sensitive foils (Tekscan, South Boston, MA, USA) with a pressure range up to 172kPa (Fig 1). These sensors feature 6 x 16 sensor cells, covering a total area of 7.6cm x 20.3cm. Thus, a detailed pressure distribution was obtained for each dedicated region. Before the measurements, conditioning, equilibration, and calibration was performed according to the manufacturer’s guidelines. Equilibration was performed at 20kPa, while three-point calibration was performed at 0kPa, 20kPa, and 50kPa. The sensors were placed on the right shoulder and right hip region of the dummy, as shown in Fig 1. It has been reported that the placement of the pressure sensors on a curved surface may induce artefacts due to bending of the sensors [37]. The signal of the sensors placed on the dummy without the load carriage system mounted was recorded prior to every measurement, but in this study, no artefacts were observed. Therefore, no further offset correction had to be conducted. The data from the pressure sensors was processed in MATLAB (R2012b, The MathWorks, Natick, MA, USA) to determine the average pressure and the peak pressure for each dedicated region. This way, the average and the peak pressure were obtained for all static and dynamic measurements in the shoulder and the hip region. With a measurement duration of 10.2 seconds at a sampling rate of 120Hz, a single measurement consisted of 1224 frames, each including pressure data of 6 x 16 sensor cells. The average pressure value of all cells interacting with the load carriage system was calculated by first taking the mean of all non-zero cells in each frame and subsequently taking the mean of these mean values over time. The peak pressure value was calculated by extracting the overall maximum value over the 6 x 16 cells and over time.

#### Strap force measurement

**Fig 3:**
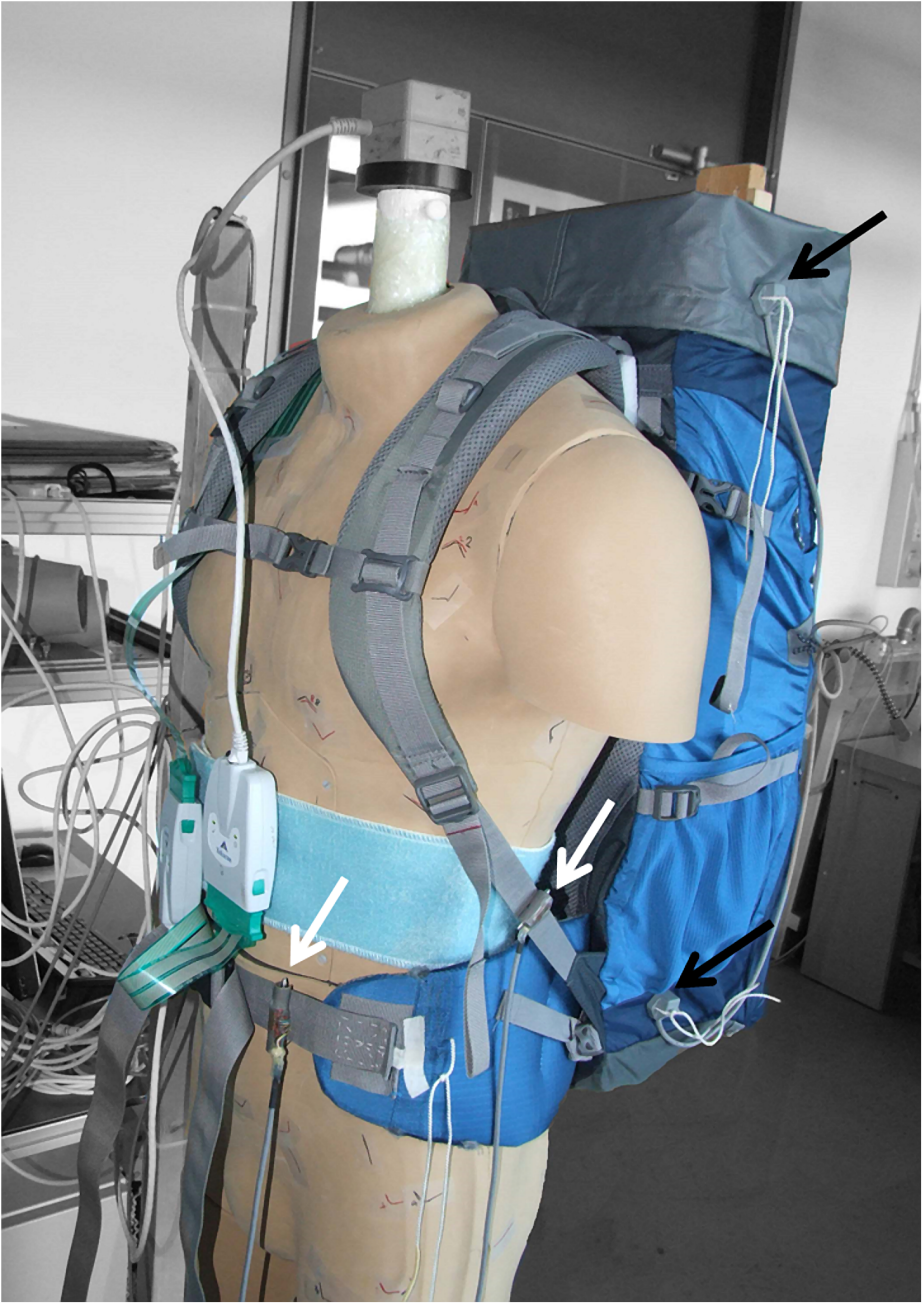
Dummy with load carriage system. The strap force sensors are mounted in the shoulder strap and the hip belt (white arrows). The Polhemus LIBERTY source is mounted on top of the dummy and its sensors are at the top and the bottom of the load carriage system (black arrows).

The forces were measured in the left shoulder strap and in the hip belt using custom made force sensors based on strain gauges (Fig 3). To calculate the strap forces for each measurement, the mean value over time was calculated.

#### Relative motion measurement

Using the 3D motion tracking system Polhemus LIBERTY 240/8 (Polhemus, Colchester, VT, USA) we measured the relative motion between the bulk of the load carriage system and the dummy. The electromagnetic field source of the tracking system was mounted on top of the dummy (Fig 3). The 6 degrees-of-freedom sensors were mounted on the left side of the load carriage system, one at the top and one at the bottom (Fig 3). For the relative motion in each measurement, the cumulative change of distance between the sensor and the source in all three axes was calculated. To normalize the relative motion over time, the cumulative change of distance was divided by the measurement duration.

### Experimental protocol

The static and the dynamic simulations on the instrumented dummy were repeated five times and the mean values are presented for all mechanical parameters. The measurement duration was 10.2 seconds for the static and the dynamic condition, resulting in 18 simulated steps (at 1.77Hz) in the dynamic measurements. For all mechanical parameters, the sampling rate was 120Hz. For each simulation, the load carriage system was removed and remounted on the dummy.

### Statistical analysis

#### Dummy validation: Correlations

For a direct validation of the dummy, we compared the mechanical parameters measured on the dummy with the same parameters measured on subjects in the previous subject study [23]. This previous subject study applied the same load carriage system with the same modifications and the same twelve configurations. For this comparison, Spearman’s rho was calculated for each mechanical parameter, indicating how well the relationship between two variables can be described using a monotonic function. A nonparametric correlation coefficient had to be chosen, as the dynamic average pressure in the hip, measured on the subjects, was not normally distributed according to the Shapiro-Wilk test (p = 0.047).

#### Predictive power of the dummy: Multiple linear regressions

We further investigated how much variation in subjectively perceived discomfort could be explained by our instrumented dummy. For the shoulder and the hip region each, a first round of multiple linear regressions was conducted with the subjective discomfort that was assessed in the previous subject study [23] as the dependent variable. The independent variables were the pressure parameters measured on the dummy, i.e. static average pressure, static peak pressure, dynamic average pressure, and dynamic peak pressure. The pressure parameters provide information independent of the type of a load carriage system, because they can be measured directly on the body surface of the dummy.

In order to investigate whether more variance in discomfort could be explained by additionally including the strap forces and the relative motion as independent variables, a second round of multiple linear regressions was performed. Due to the dependence on the type and design of a load carriage system, results including strap forces and relative motion as predictors may only be applied to load carriage systems of the specific type of backpack with a hip belt. The second round of regressions was conducted separately for the static and dynamic scenarios. This was done based on the fact that conducting multiple linear regressions with eleven degrees of freedom and eight possible predictors would not make sense, as the ratio between the number of predictors and the degrees of freedom is so poor that even with random data, a coefficient of determination R^2^ = 0.53 could be expected. All regression analyses were conducted using backwards elimination method to reduce the risk of excluding a predictor that actually does predict the outcome and therefore should not be excluded. Significance was defined as p<0.05 and Bonferroni correction for multiple testing was applied on all analyses. The choice of all statistical methods in this study was based on the guidelines and recommendations described by Field [38].

## Results

The correlation coefficients comparing the mechanical parameters between the dummy and the subjects are shown in Table 1.

**Table 1:** Correlation coefficients between the mechanical parameters measured on the subjects and on the dummy.

Parameter	Spearman-Rho
Shoulder static average pressure	0.87***
Shoulder static peak pressure	0.62*
Shoulder static strap force	0.86***
Shoulder dynamic average pressure	not significant
Shoulder dynamic peak pressure	not significant
Shoulder dynamic strap force	0.86***
Shoulder dynamic relative motion	not significant
Hip static average pressure	0.94***
Hip static peak pressure	0.86***
Hip static strap force	0.92***
Hip dynamic average pressure^+^	0.93***
Hip dynamic peak pressure	0.81**
Hip dynamic strap force	0.99***
Hip dynamic relative motion	0.87***

^+^ non-normal distribution (p = 0.047) of the subjects' variable according to Shapiro-Wilk test

* p<0.05

** p<0.01

*** p<0.001

**Fig 4:**
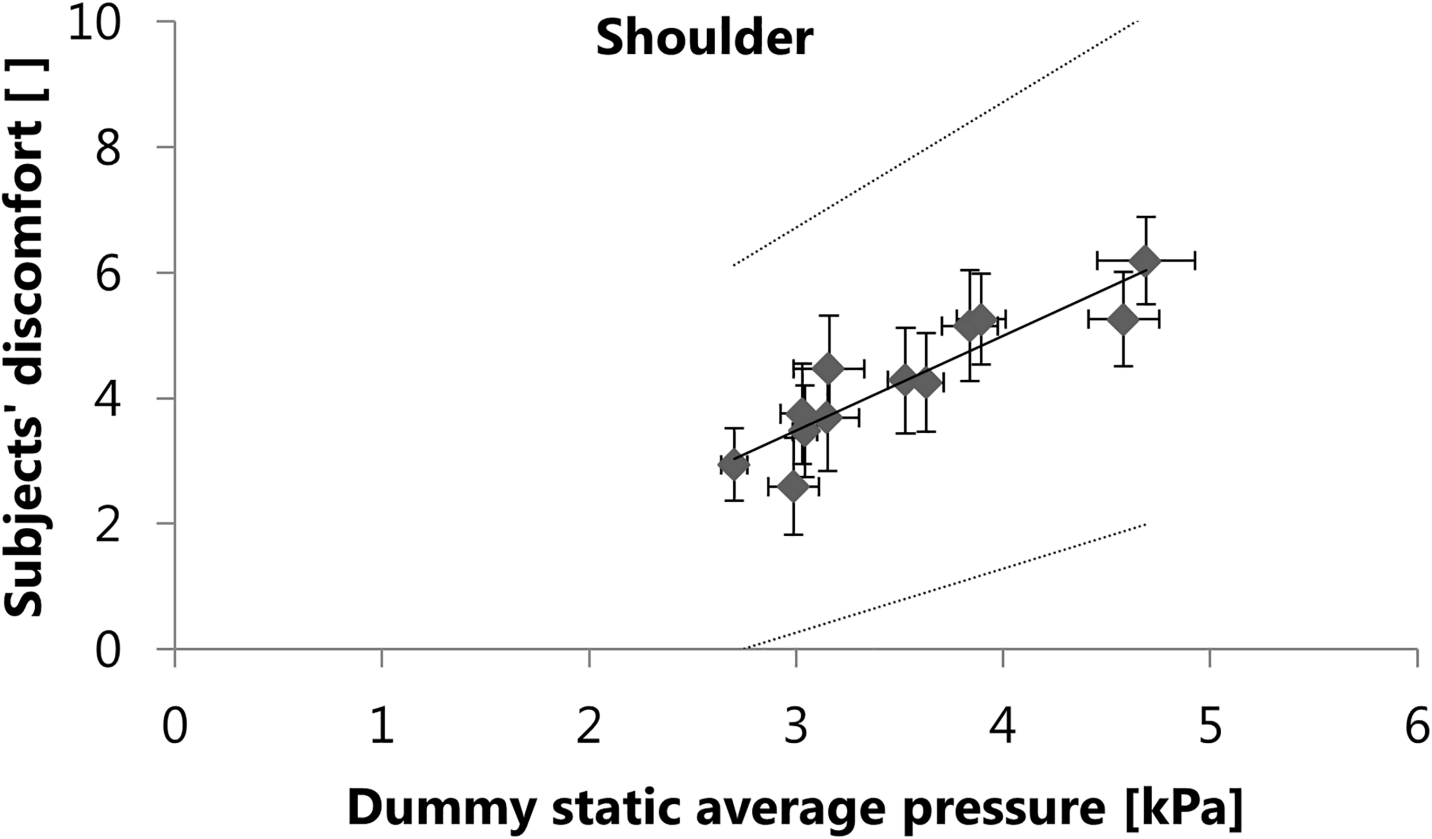
Results of the regression analysis using the pressure parameters measured on the dummy as independent variables. Static average pressure measured on the dummy is a significant (p<0.001) predictor of the subjects’ discomfort in the shoulder region. The subject’s discomfort was rated on a visual analogue scale, ranging from 0 = no discomfort to 10 = maximal discomfort. The non-significant predictors were removed from the model during the backwards elimination of the regression analysis. Data points show the mean ± standard error of measurement for each configuration, dotted lines show 95% prediction intervals. Regression equation: y = 1.512x - 1.044, R^2^ = 0.82.

**Fig 5:**
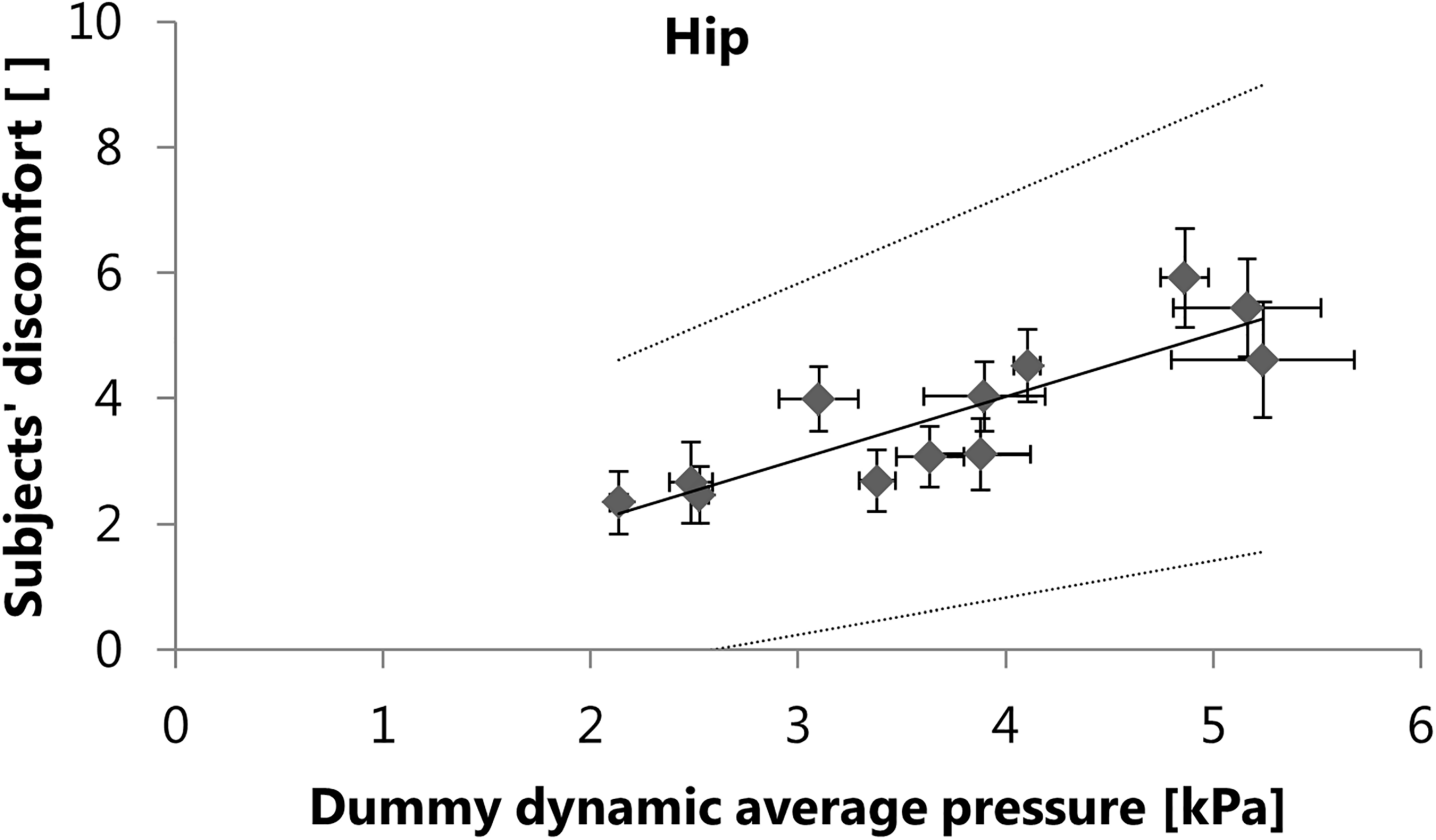
Results of the regression analysis using the pressure parameters measured on the dummy as independent variables. Dynamic average pressure measured on the dummy is a significant (p<0.001) predictor of the subjects’ discomfort in the hip region. Data points show the mean ± standard error of measurement for each configuration, dotted lines show 95% prediction intervals. Regression equation: y = 0.999x + 0.041, R^2^ = 0.75.

The result of the first round of multiple linear regression analyses, where discomfort was the dependent variable and the pressure parameters were the independent variables, is shown in Fig 4 for the shoulder region and in Fig 5 for the hip region.

The results of the second round of multiple linear regression analyses, where all mechanical parameters were entered as independent variables, are shown in Table 2. When strap force was included, the pressure parameters could not explain any significant additional variance and were excluded from the models due to the backwards elimination method (Table 2).

**Table 2:** Results of the regression analyses using average pressure, peak pressure, strap force, and relative motion measured on the dummy as independent variables.

Condition	Region	R^2^	Model	Coefficients	95% confidence interval of coefficients		Standardized coeff.
					lower	upper	
**Static**	**Shoulder**	0.88	Constant	0.855	-0.067	1.777	
			Strap force	0.116	0.086	0.146	0.938**
	**Hip**	0.79	Constant	1.425	0.505	2.346	
			Strap force	0.031	0.020	0.042	0.888**
**Dynamic**	**Shoulder**	0.90	Constant	1.032	0.252	1.812	
			Strap force	0.106	0.081	0.130	0.950**
	**Hip**	0.94	Constant	-5.429	-8.925	-1.933	
			Strap force	0.032	0.025	0.038	0.894**
			Relative motion	4.420	2.177	6.663	0.378*

* p<0.01

** p<0.001

The dependent variable was the subjects’ discomfort. Each resulting model consists of an equation to predict the subjectively perceived discomfort through a constant plus the model parameters multiplied by their coefficients. Relative motion was only entered as independent variable for the dynamic conditions.

## Discussion

In this study, we aimed to validate an instrumented dummy to assess the mechanical aspects of discomfort during load carriage by comparing the mechanical parameters that characterize the interaction between the body and the load carriage system with corresponding values from a previous subject study [23]. We further analyzed the predictive power of the instrumented dummy in assessing the discomfort of load carriage systems, applying twelve different backpack configurations.

The mechanical parameters measured on the dummy showed significant (p<0.05) correlations (Spearman’s rho>0.62) with the corresponding parameters measured on the subjects, except for the dynamic measurements in the shoulder region (Table 1). This proves the validity of the dummy for all static measurements and for dynamic measurements in the hip region to accurately simulate the interaction between the human body and the load carriage system. In the shoulder region, the pressure parameters and the relative motion revealed low agreement for the dynamic measurements. This may be due to the rigid connection of the pelvis and the shoulder girdle in the dummy, whereas the motion pattern of the subjects naturally includes a counter-rotation of the pelvis and the shoulder girdle [39–41]. Nevertheless, the shoulder strap force did correlate highly (Spearman’s rho = 0.86) in the shoulder region (Table 1). Therefore, the dynamic scenario in the shoulder region can still be assessed on the dummy with any load carriage system that feature shoulder straps and has a similar design as the system used in this study.

Regarding the predictive power of the instrumented dummy, positive results were found already for the multiple linear regression analyses with only the pressure parameters as independent variables. In the shoulder region, the static average pressure measured on the dummy can explain 82% of the variance in the subjectively perceived discomfort (Fig 4). In the hip region, the dynamic average pressure measured on the dummy can explain 75% of the variance in the subjectively perceived discomfort (Fig 5). Regarding the measurements on the dummy, average pressure is therefore a better predictor of discomfort than peak pressure, but it is not clear whether static or dynamic average pressure is more appropriate. In the shoulder region, dynamic average pressure on the dummy and on the subjects was not significantly correlated (Table 1), which makes dynamic average pressure a less suitable predictor of discomfort. In the hip region however, the correlations between the measurements on the dummy and on the subjects are similarly high for static and dynamic average pressure (Table 1). It is possible that in the absence of dynamic average pressure, static average pressure could be a significant predictor of discomfort in the hip region. Among all the mechanical parameters measured on the instrumented dummy, the pressure parameters are the ones that provide information independent of the load carriage system type, because they are measured directly on the body surface of the dummy. Therefore, the average pressure can be regarded as a significant (p<0.001) and a very powerful predictor of discomfort, regardless of the type of load carriage system evaluated.

When considering all mechanical parameters measured on the dummy, multiple linear regression analyses revealed the strap force to be a significant (p<0.001) predictor of discomfort, accounting for 79% or more of the variance in the subjectively perceived discomfort (Table 2). Strap force was more significant as a predictor than average pressure and average pressure could not explain any significant additional variance. Therefore, average pressure was excluded from the models during the backwards elimination (Table 2). For the dynamic scenario, the relative motion was also a significant (p<0.01) predictor of discomfort in the hip region. But the lower significance level of relative motion, along with its smaller standardized coefficient and the large confidence interval of its coefficient in the regression model (Table 2), indicate that strap force is more relevant. Both strap force and relative motion may depend on the type and design of a load carriage system. As all measurements in this study have been conducted with a load carriage system of a backpack with a hip belt, the relevance of strap force and relative motion as predictors of discomfort must currently be restricted to load carriage systems of comparable type and design. Most importantly, all mechanical parameters measured on the dummy that were identified as significant predictors of discomfort, are significantly (p<0.001) correlated to the corresponding parameters measured on subjects (Table 1).

Compared to the existing model of Stevenson et al. [5, 22], our instrumented dummy exhibits a higher predictive power and uses mechanical parameters as predictors that are directly validated by correlation with the same parameters from a human subject study [23]. This direct validation is one of the crucial features of the instrumented dummy presented in this study. Its uniqueness is further based on its pure focus on the mechanical aspects of discomfort. While thermo-physiological aspects play an important role in discomfort during load carriage, already existing models from the field of clothing physiology offer convenient possibilities to test load carriage systems under various climatic conditions [42, 43].

The average pressure values measured on the dummy (Figs 4 and 5) are mostly lower than the values shown to be critical for skin blood flow occlusion (5.6-16kPa), as reported by Holloway et al. [18] or Sangeorzan et al. [19]. These findings are in line with the discomfort scores not reaching the highest levels, as reported by the subjects wearing the same load carriage system configurations [23]. However, the pressure values measured on the dummy are lower than the corresponding values measured on the subjects, e.g. the static average pressure in the shoulder region ranged from 2.7kPa to 4.7kPa in the dummy measurements and from 4.5kPa to 13.5kPa in the subject study [23]. The absolute values measured on the dummy must therefore be treated with care. Because the Tekscan sensors can bend on the body surface of the subjects during dynamic measurements, the absolute values measured on the subjects may be overestimated by artefacts, despite minimizing this effect through offset correction. By contrast, the dummy has a rigid body, which holds less potential for dynamic bending of the Tekscan sensors. This may partly explain the difference between the absolute pressure values measured on the subjects and on the dummy. While the deviation from reality in terms of arm motion or torsion of the dummy is a limitation, it enables more reliable and more objective measurements than subject studies. Compared to subject studies, the dummy is also more objective in case of potentially confounding factors. Effects of the aesthetic design of a load carriage system or personal moods are excluded. Due to the reproducible test conditions, future measurements of load carriage systems can also be directly compared with existing data, resulting from previous measurements on the instrumented dummy.

Possible improvements of the instrumented dummy include introducing a torsional movement in the transversal plane to perform counter-rotation of the pelvis and the shoulder girdle and thus achieve a more complex but also more realistic representation of human walking during the dynamic measurements. Complexity may also be added in the simulated skin and soft tissue layer on the body surface of the dummy. However, any change towards a thicker or softer layer in places where the pressure distribution will be assessed is likely to result in a reduced test-retest reliability due to bending of the pressure sensors. An obvious improvement would be to offer an additional dummy with female anatomy. Gender specific differences in the mechanical parameters and in the perception of discomfort during load carriage are likely and express a need for future work focusing on female subjects and dummies with female anatomy. As a complete alternative to a mechanical dummy, finite element models may also need to be considered. Existing models, such as the validated Global Human Body Modeling 50th percentile male model [44] could provide an excellent possibility to study the interaction between load carriage systems and the human body. However, this would likely increase the cost of testing and comparing large numbers of load carriage systems, as for each system, a separate finite element model would have to be created and validated. Generally, the validation of the instrumented dummy presented in this study reduces the need for subject studies to assess discomfort during load carriage. The dummy enables objective, fast, and iterative assessments during the development of new designs of load carriage systems as well as comparisons of many different systems or designs. Such an objective assessment may also offer a basis for a simple rating system as it is already widely used in the automotive industry, in the form of a 5-star safety rating.

### Limitations

The body surface regions investigated in this study are currently limited to the shoulder and hip region, as they are considered to be the most critical regarding discomfort during load carriage. To allow the application of our method to all relevant body regions, further measurements may be necessary. In addition, the dummy represents a simplified approximation of the male human body and does not feature variation in the hardness of its body surface depending on the body region. Furthermore, the forward leaning angles of the dummy are calculated based on the assumption of balancing the moment in the upper ankle joint and possible adaptations in other joints are neglected.

## Conclusions

In this study, an objective and time-saving method to assess or compare the discomfort of load carriage systems and their design has been validated. The instrumented dummy can explain 75% or more of the variance in discomfort using average pressures as predictors and even 79% or more of the variance in discomfort using strap forces as predictors. Compared to an existing model [5], our model has a higher predictive power and it uses mechanical parameters as predictors that are directly validated by correlation with the same parameters measured in a human subject study [23]. Our model enables objective, fast, and iterative assessments during the development of new designs of load carriage systems as well as comparisons of many different systems or designs. As a consequence, the need for subject studies is reduced and users of backpacks and other load carriage systems may profit from future load carriage systems that inflict less discomfort during load carriage.

## Supporting information

S1 Table Mechanical parameters in the shoulder region of the instrumented dummy. For each measured configuration, the mean of five iterations ± the standard error of measurement is shown.
(DOCX)

S2 Table Mechanical parameters in the hip region of the instrumented dummy. For each measured configuration, the mean of five iterations ± the standard error of measurement is shown.
(DOCX)
