# Supplementary material for: Validation of an instrumented dummy to assess mechanical aspects of discomfort during load carriage
Source: PLoS One. 2017 Jun 29;12(6):e0180069. doi: 10.1371/journal.pone.0180069 (PMC5491328; doi:10.1371/journal.pone.0180069)
Supplement: S1 Table — For each measured configuration, the mean of five iterations ± the standard error of measurement is shown. (DOCX) [file pone.0180069.s001.docx]

**S1 Table.** **Mechanical parameters in the shoulder region.**

| **Shoulder region** | **Average pressure [kPa]** | | **Peak pressure [kPa]** | | **Strap force [N]** | | **Relative motion [mm/s]** |
| --- | --- | --- | --- | --- | --- | --- | --- |
| **Configuration *** | static | dynamic | static | dynamic | static | dynamic | dynamic |
| **1** (15.0 kg, 30 N) | 3.5 ± 0.1 | 3.6 ± 0.1 | 22.5 ± 0.7 | 23.9 ± 0.6 | 32.0 ± 1.0 | 32.0 ± 0.8 | 1.45 ± 0.07 |
| **2** (15.0 kg, 60 N) | 3.0 ± 0.1 | 3.1 ± 0.1 | 15.7 ± 0.7 | 17.0 ± 0.6 | 27.3 ± 1.1 | 27.0 ± 0.9 | 1.41 ± 0.03 |
| **3** (15.0 kg, 90 N) | 2.7 ± 0.1 | 2.7 ± 0.1 | 11.3 ± 0.7 | 12.1 ± 0.6 | 22.1 ± 0.8 | 21.8 ± 0.9 | 1.49 ± 0.05 |
| **4** (15.0 kg, 120 N) | 3.0 ± 0.1 | 2.8 ± 0.1 | 14.6 ± 0.7 | 14.7 ± 0.6 | 17.1 ± 0.8 | 17.5 ± 0.7 | 1.73 ± 0.01 |
| **5** (20.0 kg, 30 N) | 3.8 ± 0.1 | 3.9 ± 0.1 | 20.0 ± 1.2 | 22.0 ± 1.3 | 33.0 ± 1.6 | 34.0 ± 1.3 | 1.89 ± 0.07 |
| **6** (20.0 kg, 60 N) | 3.6 ± 0.1 | 3.5 ± 0.2 | 20.3 ± 1.0 | 21.0 ± 0.9 | 28.2 ± 1.2 | 29.4 ± 0.8 | 1.92 ± 0.06 |
| **7** (20.0 kg, 90 N) | 3.2 ± 0.1 | 2.9 ± 0.1 | 16.2 ± 1.3 | 15.6 ± 1.0 | 24.3 ± 1.1 | 24.5 ± 0.8 | 2.41 ± 0.14 |
| **8** (20.0 kg, 120 N) | 3.0 ± 0.1 | 2.9 ± 0.1 | 15.7 ± 1.2 | 16.1 ± 1.2 | 19.7 ± 0.8 | 20.5 ± 0.8 | 2.35 ± 0.11 |
| **9** (25.0 kg, 30 N) | 4.7 ± 0.2 | 4.2 ± 0.3 | 23.8 ± 2.0 | 21.3 ± 1.5 | 48.1 ± 1.5 | 51.2 ± 1.7 | 3.02 ± 0.07 |
| **10** (25.0 kg, 60 N) | 4.6 ± 0.2 | 4.6 ± 0.1 | 23.2 ± 0.5 | 23.2 ± 0.7 | 37.8 ± 1.0 | 40.7 ± 0.6 | 3.20 ± 0.03 |
| **11** (25.0 kg, 90 N) | 3.9 ± 0.1 | 3.6 ± 0.1 | 19.0 ± 0.5 | 19.1 ± 0.6 | 35.3 ± 1.7 | 37.5 ± 1.3 | 3.13 ± 0.03 |
| **12** (25.0 kg, 120 N) | 3.2 ± 0.2 | 2.4 ± 0.1 | 13.4 ± 0.7 | 14.2 ± 0.3 | 29.5 ± 1.7 | 31.5 ± 1.0 | 3.28 ± 0.07 |

For each measured configuration, the mean of five iterations ± the standard error of measurement is shown.

* The configurations differ in load mass and tension to which the hip belt was adjusted, as shown in brackets.
