# Supplementary material for: Validation of an instrumented dummy to assess mechanical aspects of discomfort during load carriage
Source: PLoS One. 2017 Jun 29;12(6):e0180069. doi: 10.1371/journal.pone.0180069 (PMC5491328; doi:10.1371/journal.pone.0180069)
Supplement: S2 Table — For each measured configuration, the mean of five iterations ± the standard error of measurement is shown. (DOCX) [file pone.0180069.s002.docx]

**S2 Table.** **Mechanical parameters in the hip region.**

| **Hip region** | **Average pressure [kPa]** | | **Peak pressure [kPa]** | | **Strap force [N]** | | **Relative motion [mm/s]** |
| --- | --- | --- | --- | --- | --- | --- | --- |
| **Configuration *** | static | dynamic | static | dynamic | static | dynamic | dynamic |
| **1** (15.0 kg, 30 N) | 2.2 ± 0.1 | 2.1 ± 0.1 | 9.0 ± 0.3 | 9.0 ± 0.3 | 30.5 ± 0.2 | 31.1 ± 0.2 | 1.46 ± 0.01 |
| **2** (15.0 kg, 60 N) | 3.4 ± 0.1 | 3.4 ± 0.1 | 13.5 ± 0.2 | 13.6 ± 0.2 | 60.2 ± 0.3 | 60.3 ± 0.3 | 1.41 ± 0.01 |
| **3** (15.0 kg, 90 N) | 3.9 ± 0.2 | 3.9 ± 0.2 | 18.6 ± 1.3 | 18.8 ± 1.4 | 90.2 ± 0.3 | 89.5 ± 0.3 | 1.42 ± 0.01 |
| **4** (15.0 kg, 120 N) | 5.5 ± 0.5 | 5.2 ± 0.4 | 27.2 ± 2.5 | 27.6 ± 2.5 | 119.5 ± 0.2 | 119.1 ± 0.4 | 1.42 ± 0.01 |
| **5** (20.0 kg, 30 N) | 2.3 ± 0.1 | 2.5 ± 0.1 | 10.4 ± 0.6 | 10.9 ± 0.6 | 30.8 ± 0.1 | 31.8 ± 0.2 | 1.51 ± 0.01 |
| **6** (20.0 kg, 60 N) | 3.6 ± 0.2 | 3.6 ± 0.2 | 20.8 ± 2.1 | 20.9 ± 2.1 | 60.1 ± 0.2 | 60.3 ± 0.4 | 1.50 ± 0.02 |
| **7** (20.0 kg, 90 N) | 3.8 ± 0.3 | 3.9 ± 0.3 | 25.2 ± 1.8 | 25.8 ± 1.9 | 89.6 ± 0.3 | 89.8 ± 0.2 | 1.52 ± 0.01 |
| **8** (20.0 kg, 120 N) | 5.2 ± 0.4 | 5.2 ± 0.4 | 27.7 ± 2.2 | 28.1 ± 2.1 | 119.5 ± 0.2 | 118.5 ± 0.3 | 1.56 ± 0.02 |
| **9** (25.0 kg, 30 N) | 2.4 ± 0.1 | 2.5 ± 0.1 | 11.5 ± 0.6 | 12.6 ± 0.6 | 30.6 ± 0.2 | 31.9 ± 0.3 | 1.68 ± 0.01 |
| **10** (25.0 kg, 60 N) | 3.0 ± 0.2 | 3.1 ± 0.2 | 13.5 ± 1.1 | 14.2 ± 1.1 | 59.9 ± 0.3 | 60.6 ± 0.4 | 1.68 ± 0.00 |
| **11** (25.0 kg, 90 N) | 4.0 ± 0.1 | 4.1 ± 0.1 | 16.9 ± 0.4 | 17.9 ± 0.3 | 89.8 ± 0.1 | 89.5 ± 0.3 | 1.62 ± 0.00 |
| **12** (25.0 kg, 120 N) | 5.0 ± 0.1 | 4.9 ± 0.1 | 21.7 ± 1.0 | 22.0 ± 0.7 | 119.6 ± 0.3 | 119.1 ± 0.3 | 1.66 ± 0.01 |

For each measured configuration, the mean of five iterations ± the standard error of measurement is shown.

* The configurations differ in load mass and tension to which the hip belt was adjusted, as shown in brackets.
